# Comparative Effectiveness and Safety of Denosumab Versus Bisphosphonates in Elderly Patients with Cancer Bone Metastases: A Target Trial Emulation Study

**DOI:** 10.3390/life16020346

**Published:** 2026-02-17

**Authors:** Che-Wei Liu, Shun-Neng Hsu, Shao-Hsuan Chang, Wei-Cheng Chang, Chun-Liang Hsu, Hsin-Yu Chen, Po-Huang Chen, Cho-Hao Lee

**Affiliations:** 1 Department of Orthopaedics, Cathay General Hospital, Taipei 10630, Taiwan; petergates38@gmail.com; 2 School of Medicine, College of Medicine, Fu Jen Catholic University, New Taipei City 242062, Taiwan; 3 School of Medicine, National Tsing Hua University, Hsinchu 300044, Taiwan; 4 Division of Nephrology, Department of Internal Medicine, Tri-Service General Hospital, National Defense Medical University, Taipei 114202, Taiwan; h720127@gmail.com; 5 Department of Internal Medicine, School of Medicine, College of Medicine, National Defense Medical University, Taipei 11258, Taiwan; 6 Department of Biomedical Engineering, Chang Gung University, Taoyuan 333323, Taiwan; 7 Department of Ophthalmology, Universe Eye Center, Taipei 115, Taiwan; 8 Orthopaedic Department, Tri-Service General Hospital, National Defense Medical University, Taipei 114202, Taiwan; windoverture@gmail.com; 9 Department of Family Medicine, Chi Mei Medical Center, Tainan 710, Taiwan; 10 Division of Hematology and Oncology, Department of Internal Medicine, Tri-Service General Hospital, National Defense Medical University, Taipei 114202, Taiwan; 11 Department of Oncology, Tri-Service General Hospital, National Defense Medical University, Taipei 114202, Taiwan

**Keywords:** bone metastases, Denosumab, bisphosphonates, skeletal-related events, target trial emulation

## Abstract

**Objective:** Bone-modifying agents (BMA) are central to the prevention of skeletal-related events (SREs) in patients with cancer bone metastases, yet evidence guiding agent selection in very old patients remains limited. This study aimed to compare the effectiveness and safety of Denosumab versus bisphosphonates in patients aged ≥75 years with solid tumour-related bone metastases using a target trial emulation framework. **Methods:** We conducted a retrospective cohort study using the TriNetX Global Collaborative Network to emulate a hypothetical randomised trial. Patients aged ≥75 years with solid tumour-related bone metastases initiating Denosumab or bisphosphonates were included. After 1:1 propensity score matching (PSM), 10,662 patients were analysed in each treatment group. The primary outcome was time to first SRE. Secondary outcomes included individual SRE components, all-cause mortality, and safety events. **Results:** Among 21,324 matched patients (mean age, 75.6 years), bisphosphonate use was associated with a higher risk of SREs compared with Denosumab (hazard ratio [HR], 1.15; 95% CI, 1.06–1.25). The excess risk was driven by pathological fractures (HR, 1.28; 95% CI, 1.10–1.49), whereas other SRE components did not differ significantly. All-cause mortality was higher among bisphosphonate users (HR, 1.41; 95% CI, 1.33–1.49, *p* < 0.001). Hypocalcaemia occurred more frequently with Denosumab (5.7% vs. 2.4%), while risks of acute kidney injury and end-stage renal disease (ESRD) were similar. Findings were consistent across sensitivity and subgroup analyses. **Conclusions**: In patients aged ≥75 years with solid tumour-related bone metastases, Denosumab was associated with lower risks of skeletal-related events—particularly pathological fractures—and reduced all-cause mortality compared with bisphosphonates. These results extend randomised trial evidence to a clinically vulnerable population and support Denosumab as a preferred BMA in older adults.

## 1. Introduction

Bone metastases represent a significant clinical burden in patients with advanced solid tumours, affecting approximately 70% of patients with breast and prostate cancer and 30–40% of patients with lung, kidney, and other cancers [1,2]. The presence of bone metastases leads to substantial morbidity through skeletal-related events (SREs), which include pathological fractures, spinal cord compression, need for radiation therapy to bone, and surgical intervention [3]. These complications not only impair quality of life but also increase healthcare costs and may negatively impact survival [4].

As such, bone-modifying agents (BMAs) have become the standard of care for preventing SREs in patients with bone metastases. Two main classes of BMAs are currently available: bisphosphonates (primarily zoledronic acid) and the RANK ligand inhibitor denosumab [5]. Bisphosphonates and RANKL inhibitors reduce skeletal-related events through distinct but complementary antiresorptive mechanisms. Nitrogen-containing bisphosphonates (e.g., zoledronic acid) bind to hydroxyapatite at sites of active bone remodelling and are internalised by osteoclasts during bone resorption. They inhibit farnesyl pyrophosphate synthase in the mevalonate pathway, leading to impaired prenylation of small GTPases, osteoclast dysfunction, and ultimately apoptosis. This mechanism results in a long skeletal half-life due to strong bone matrix binding. In contrast, Denosumab is a fully human monoclonal antibody that binds to and neutralises RANKL (Receptor Activator of Nuclear Factor-κB Ligand). By mimicking the effect of osteoprotegerin, Denosumab prevents the maturation and activation of osteoclast precursors, thereby suppressing bone resorption upstream of the stage at which bisphosphonates act.

Three pivotal randomised controlled trials (RCTs) comparing Denosumab to zoledronic acid in patients with bone metastases from breast cancer, prostate cancer, and other solid tumours demonstrated the superiority of Denosumab in delaying time to first SRE, with hazard ratios (HR) ranging from 0.82 to 0.84 [6,7,8]. However, these landmark trials predominantly enrolled younger patients, with mean ages ranging from 57 to 71 years, and patients with significant renal impairment were often excluded. Older patients represent a unique population with distinct pharmacokinetic profiles, higher comorbidity burden, and different risk–benefit considerations. Bisphosphonates require renal dose adjustment and are contraindicated in severe renal impairment, while Denosumab does not require renal dose adjustment but carries a higher risk of hypocalcaemia [9,10]. The comparative effectiveness and safety of these agents in real-world older populations remain inadequately characterised.

Target trial emulation provides a rigorous framework for using observational data to estimate causal effects that would be obtained from a hypothetical randomised trial [11,12]. This approach explicitly specifies the target trial protocol and then emulates each component using observational data, thereby reducing confounding and various biases inherent in traditional observational studies.

The objective of this study was to emulate a target trial comparing the effectiveness and safety of Denosumab versus bisphosphonates in older patients (aged ≥75 years) with solid-tumour bone metastases, using real-world data from a large federated electronic health record network.

## 2. Methods

### 2.1. Study Design and Data Source

We conducted a retrospective cohort study using the TriNetX Global Collaborative Network, a federated health research platform that provides access to de-identified electronic health records from over 193 million patients across healthcare organisations worldwide. The network includes comprehensive longitudinal data on diagnoses, procedures, medications, laboratory values, and vital status. This study utilised a target trial emulation framework with propensity score matching (PSM) to compare the effectiveness and safety of Denosumab versus bisphosphonates.

### 2.2. Study Population

The study population included patients aged ≥75 years with a diagnosis of solid tumour malignancy (ICD-10-CM: C00-C80, C7A, C7B) and secondary malignant neoplasm of bone (ICD-10-CM: C79.51, C79.52) who initiated a BMA after their bone metastasis diagnosis. Patients were required to have at least 12 months of healthcare records prior to the index date to ensure adequate baseline assessment. Exclusion criteria included: (1) multiple myeloma (ICD-10-CM: C90), given its different pathophysiology; (2) prior use of the comparator drug to ensure a treatment-naïve comparison; (3) SREs within one year before the index date to focus on prevention rather than treatment; (4) estimated glomerular filtration rate (eGFR) < 15 mL/min/1.73 m^2^ or end-stage renal disease (ESRD), as bisphosphonates are contraindicated in severe renal impairment; and (5) history of drug-induced osteonecrosis of the jaw.

### 2.3. Exposure Definition

The bisphosphonate cohort included patients initiating zoledronic acid (RxNorm: 77655), pamidronate (RxNorm: 11473), or clodronic acid (RxNorm: 3350). The denosumab cohort included patients initiating Denosumab at oncology dosing (RxNorm: 993449; HCPCS: J0897). The index date was defined as the date of the first BMA prescription after bone metastasis diagnosis. Patients who had previously received the comparator drug were excluded to ensure new-user status.

### 2.4. Outcomes

The primary outcome was SRE, defined as a composite of (1) pathological fracture in neoplastic disease (ICD-10-CM: M84.5x); (2) spinal cord compression (ICD-10-CM: G95.2, G55); (3) radiation therapy to bone (CPT: 77401–77425, 77385–77386); (4) surgery to bone including vertebroplasty and kyphoplasty (CPT: 22520–22525); and functional impairment (Z74.01). This definition aligns with SRE endpoints used in pivotal clinical trials [6,7,8]. Secondary outcomes included individual SRE components related to bone metastases and all-cause mortality. Safety outcomes included acute kidney injury (ICD-10-CM: N17.x), hypocalcaemia (ICD-10-CM: E83.51), and progression to ESRD (ICD-10-CM: N18.6, Z99.2). The outcome window extended from 30 days to 365 days after the index date.

### 2.5. Covariates

Baseline covariates assessed during the 12 months prior to the index date included: demographics (age, sex, race, facility type); primary cancer type (breast, prostate, lung, kidney, other); comorbidities (diabetes mellitus, heart failure, chronic kidney disease [CKD]); concomitant medications (corticosteroids, calcium supplements, antineoplastic agents, endocrine therapy); and laboratory values (body mass index, creatinine, alkaline phosphatase, eGFR).

### 2.6. Statistical Analysis

Propensity scores were estimated using logistic regression, including all baseline covariates. A 1:1 nearest-neighbour matching was performed using a greedy algorithm with a calliper width of 0.1 standard deviation of the logit of the propensity score. Covariate balance was assessed using standardised mean differences (SMD), with SMD < 0.1 considered indicative of adequate balance [13]. Time-to-event outcomes were analysed using Kaplan–Meier survival analysis with log-rank tests and Cox proportional hazards regression. To account for the non-independence of the matched observations, the Cox models were stratified on the matched pairs. HR with 95% confidence intervals (CI) were calculated, with Denosumab as the reference group. E-values were computed to assess the robustness of findings to potential unmeasured confounding [14]. Sensitivity analyses included: (1) restriction to the US population only; (2) expanded age criterion (≥65 years); and (3) per-protocol analysis requiring continuous exposure (BMA ≥ 6 medical records). Subgroup analyses were performed by age strata (75–79, 80–84, ≥85 years), race, cancer type, eGFR level, and presence of comorbidities. Negative control outcomes (cholelithiasis, nephrolithiasis, burns) were analysed to detect residual confounding. All analyses were performed using the TriNetX platform (https://trinetx.com/). A two-sided *p* < 0.05 was considered statistically significant.

### 2.7. Ethical Considerations

This study used de-identified data from the TriNetX network and was therefore exempt from institutional review board approval. The study was conducted in accordance with the Declaration of Helsinki.

## 3. Results

### 3.1. Study Population

**Figure 1:**
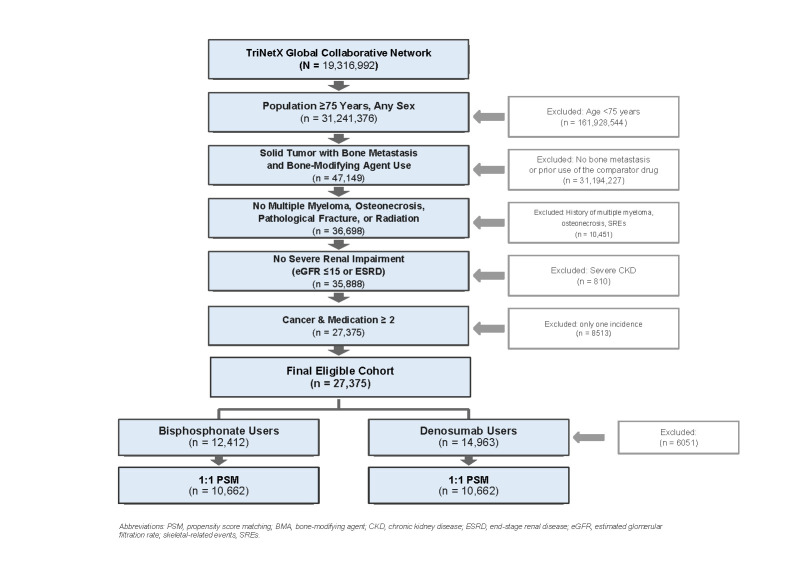
Patient selection flowchart. Sequential application of inclusion and exclusion criteria from the TriNetX Global Collaborative Network to derive the final propensity score-matched cohort. Abbreviations: PSM, propensity score matching; BMA, bone-modifying agent; CKD, chronic kidney disease; ESRD, end-stage renal disease.

From the TriNetX Global Collaborative Network of 193,169,920 patients, we identified 31,241,376 patients aged ≥75 years. After applying the inclusion criteria for solid tumour with bone metastasis and BMA use, 47,149 patients remained. Following exclusion of patients with multiple myeloma, prior osteonecrosis, prior SRE, and severe renal impairment, the final eligible cohort comprised 27,375 patients (12,412 bisphosphonate users and 14,963 denosumab users). After 1:1 PSM, 10,662 patients remained in each treatment group (Figure 1).

### 3.2. Baseline Characteristics

Before matching, there were notable differences between the treatment groups. Denosumab users were older (76.7 ± 6.6 vs. 74.9 ± 6.9 years), more likely to have prostate cancer (41.1% vs. 30.1%), and more likely to receive endocrine therapy (46.6% vs. 33.4%). After PSM, all baseline characteristics were well-balanced with SMD < 0.1 for most variables (Table 1). In the matched cohort, the mean age was 75.6 years in both groups. The most common primary cancers were prostate (33.4%), breast (22.8–23.1%), and lung (19.4–19.6%). Comorbidities included diabetes mellitus (18.0–18.2%), CKD (11.5–11.8%), and heart failure (7.7–8.0%). Baseline mean eGFR was 74.0 mL/min/1.73 m^2^ in the bisphosphonate group and 71.8 mL/min/1.73 m^2^ in the denosumab group. Notably, key renal parameters, including the prevalence of chronic kidney disease and baseline eGFR levels, were well-balanced between the two groups after matching (all SMDs < 0.1), ensuring the comparability of the cohorts regarding renal safety and related mortality risks.

**Table 1:** Baseline Characteristics Before and After Propensity Score Matching.

	Before Matching			After Matching		
	Bisphosphonates	Denosumab	SMD	Bisphosphonates	Denosumab	SMD
	N = 12,412	N = 14,963		N = 10,662	N = 10,662	
**Demographics**						
Age at Index, years (mean ± SD)	74.9 ± 6.9	76.7 ± 6.6	0.255	75.6 ± 6.8	75.6 ± 6.4	0.004
Male, n (%)	6933 (57.5)	8981 (60.0)	0.051	6060 (56.8)	6094 (57.2)	0.006
Female, n (%)	5116 (42.5)	5980 (40.0)	0.051	4601 (43.2)	4567 (42.8)	0.006
**Race**						
White, n (%)	8340 (72.2)	10,808 (74.9)	0.066	7551 (73.7)	7614 (74.2)	0.013
Black or African American, n (%)	976 (8.5)	1179 (8.2)	0.008	831 (8.1)	782 (7.6)	0.017
Asian, n (%)	1003 (8.7)	1216 (8.4)	0.007	862 (8.4)	859 (8.4)	0.001
**Diagnosis**						
Breast cancer, n (%)	2645 (22.0)	3395 (22.7)	0.018	2431 (22.8)	2460 (23.1)	0.006
Prostate cancer, n (%)	3625 (30.1)	6148 (41.1)	0.231	3559 (33.4)	3561 (33.4)	<0.001
Lung cancer, n (%)	2525 (21.0)	2459 (16.4)	0.116	2085 (19.6)	2069 (19.4)	0.004
Kidney cancer, n (%)	476 (4.0)	544 (3.6)	0.016	419 (3.9)	402 (3.8)	0.008
Diabetes mellitus, n (%)	2260 (18.8)	2661 (17.8)	0.025	1945 (18.2)	1924 (18.0)	0.005
Heart failure, n (%)	998 (8.3)	1153 (7.7)	0.021	848 (8.0)	819 (7.7)	0.010
CKD, n (%)	1371 (11.4)	1961 (13.1)	0.053	1263 (11.8)	1224 (11.5)	0.011
**Laboratory**						
BMI, kg/m^2^ (mean ± SD)	26.6 ± 5.9	26.9 ± 5.6	0.040	26.7 ± 5.8	26.8 ± 5.7	0.012
eGFR, mL/min/1.73 m^2^ (mean ± SD)	74.8 ± 28.4	70.8 ± 26.4	0.147	74.0 ± 27.7	71.8 ± 26.6	0.078
Creatinine, mg/dL (mean ± SD)	1.1 ± 4.5	1.1 ± 0.5	0.022	1.2 ± 4.8	1.0 ± 0.4	0.037
ALP, U/L (mean ± SD)	181.3 ± 274.6	160.0 ± 266.1	0.079	181.7 ± 282.3	175.1 ± 228.3	0.013

Abbreviations: 1. patients (10.7%) in the bisphosphonate group and 1122 patients (10.5%) in the denosumab group. Bisphosphonate use was associated with a significantly higher risk of SRE compared to Denosumab (HR 1.15, 95% CI: 1.06–1.25, *p* = 0.001). The E-value for this finding was 1.57, indicating moderate robustness to unmeasured confounding (Table 2). Kaplan–Meier survival curves demonstrated early and persistent separation between treatment groups, with bisphosphonate users experiencing faster time to first SRE (Figure 2).

**Table 2:** Clinical Outcomes After Propensity Score Matching.

Outcome	Bisphosphonates Events, n (%)	Denosumab Events, n (%)	HR (95% CI)	*p* Value	E-Value
**Skeletal-Related Events**	1146 (10.7)	1122 (10.5)	1.15 (1.06–1.25)	0.001	1.57
Pathological fracture	340 (3.2)	310 (2.9)	1.28 (1.10–1.49)	<0.001	1.88
Spinal cord compression	55 (0.5)	50 (0.5)	1.29 (0.88–1.89)	0.192	1.91
Radiation therapy to bone	635 (6.0)	650 (6.1)	1.10 (0.99–1.23)	0.082	1.42
Vertebral surgery	24 (0.2)	27 (0.3)	1.04 (0.60–1.81)	0.882	1.21
Functional impairment	92 (0.9)	85 (0.8)	1.27 (0.94–1.71)	0.118	1.85
**All-cause mortality**	2631 (24.7)	2258 (21.2)	1.41 (1.33–1.49)	<0.001	2.17
**Safety Outcomes**					
Acute kidney injury	886 (8.3)	1031 (9.7)	1.01 (0.92–1.11)	0.816	1.11
Hypocalcaemia	261 (2.4)	609 (5.7)	0.49 (0.43–0.57)	<0.001	3.50
ESRD	21 (0.2)	40 (0.4)	0.63 (0.37–1.06)	0.079	2.55

N 10 per group. Reference group: Denosumab. Data are presented as n (%) for events. Abbreviations: 2. Kaplan–Meier survival curves for skeletal-related events. Panel A shows the unmatched cohorts; Panel B shows results after 1:1 PSM. Numbers at risk are displayed at 60-day intervals. Shaded areas represent 95% confidence intervals. HR, hazard ratio; CI, confidence interval.

**Figure 2:**
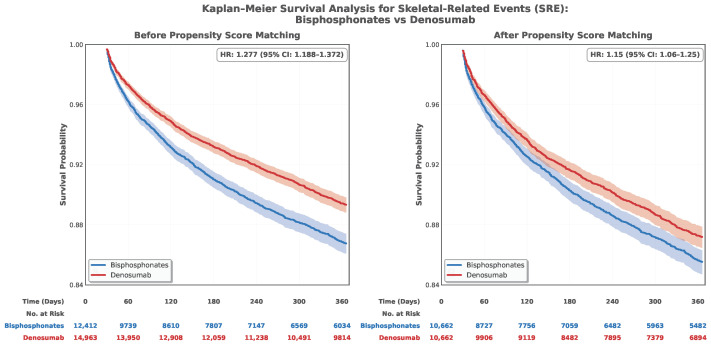
Kaplan-Meier survival analysis for skeletal-related events.

### 3.3. Secondary Outcomes

Analysis of individual SRE components demonstrated that, compared with Denosumab, bisphosphonate use was associated with a significantly higher risk of pathological fracture (HR 1.28, 95% CI: 1.10–1.49; *p* < 0.001). In contrast, no statistically significant differences were observed between groups for spinal cord compression (HR 1.29, 95% CI: 0.88–1.89; *p* = 0.192), radiation therapy to bone (HR 1.10, 95% CI: 0.99–1.23; *p* = 0.082), vertebral surgery (HR 1.04, 95% CI: 0.60–1.81; *p* = 0.882), or functional impairment (HR 1.27, 95% CI: 0.94–1.71; *p* = 0.118). All-cause mortality was significantly higher among bisphosphonate users, with 2631 deaths (24.7%) compared with 2258 deaths (21.2%) in the denosumab group (HR 1.41, 95% CI: 1.33–1.49; *p* < 0.001). The corresponding E-value of 2.17 suggests moderate-to-strong robustness of this association to potential unmeasured confounding (Table 2).

### 3.4. Safety Outcomes

Acute kidney injury occurred in 886 patients (8.3%) in the bisphosphonate group and 1031 patients (9.7%) in the denosumab group, with no significant difference between groups (HR 1.01, 95% CI: 0.92–1.11, *p* = 0.816). This finding was unexpected, given the known nephrotoxicity of bisphosphonates, potentially reflecting appropriate patient selection and dose adjustment in clinical practice. Hypocalcaemia was significantly more common in the denosumab group, occurring in 609 patients (5.7%) compared with 261 patients (2.4%) in the bisphosphonate group (HR 0.49, 95% CI: 0.43–0.57, *p* < 0.001), indicating a lower risk with bisphosphonates. This finding is consistent with the known pharmacologic effect of Denosumab on calcium homeostasis. No significant difference was observed for ESRD between groups (HR 0.63, 95% CI: 0.37–1.06, *p* = 0.079).

### 3.5. Sensitivity Analyses

Sensitivity analyses consistently supported the primary findings. In the US-only population (N = 8396 per group), bisphosphonates remained associated with higher SRE risk (HR 1.18, 95% CI: 1.08–1.28, *p* < 0.001) and mortality (HR 1.41, 95% CI: 1.32–1.50, *p* < 0.001). In the expanded age cohort (≥65 years, N = 17,252 per group), similar patterns were observed for SRE (HR 1.15, 95% CI: 1.08–1.22, *p* < 0.001) and mortality (HR 1.43, 95% CI: 1.37–1.49, *p* < 0.001). The per-protocol analysis (N = 3381 per group) also confirmed higher SRE risk with bisphosphonates (HR 1.23, 95% CI: 1.08–1.42, *p* = 0.003) (Appendix S5 in Supplementary Materials).

### 3.6. Subgroup Analyses

**Figure 3:**
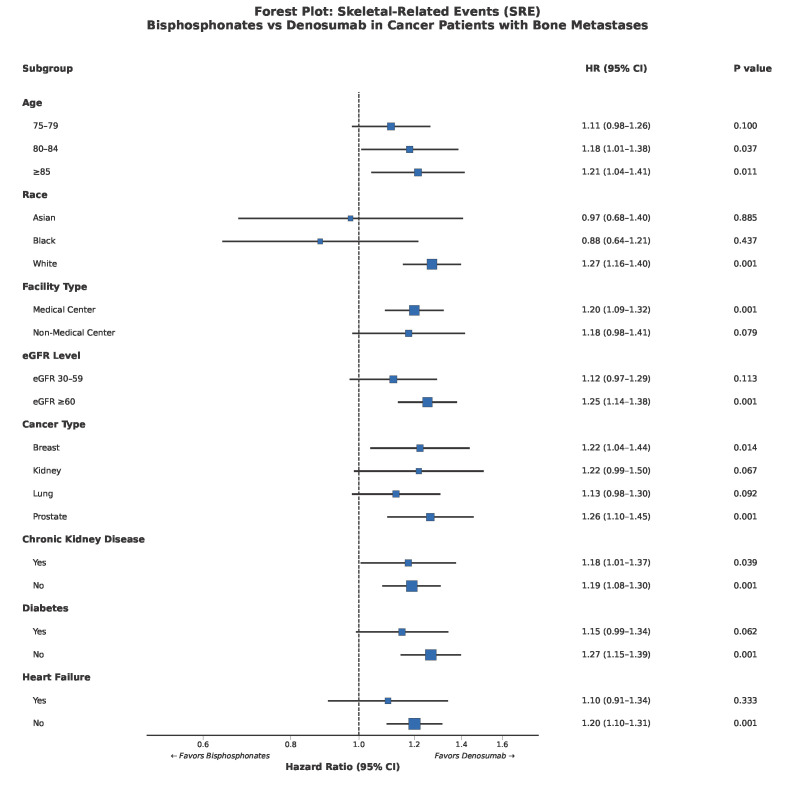
Forest plot of subgroup analyses for skeletal-related events. Hazard ratios with 95% confidence intervals are shown for prespecified subgroups. The dashed vertical line represents HR = 1.0 (no difference). HR > 1.0 favours Denosumab. Abbreviations: CKD, chronic kidney disease; DM, diabetes mellitus; HF, heart failure; eGFR, estimated glomerular filtration rate.

Subgroup analyses demonstrated generally consistent treatment effects across patient subgroups (Figure 3). The benefit of Denosumab over bisphosphonates for SRE prevention was statistically significant in patients aged ≥85 years (HR 1.21, 95% CI: 1.04–1.41, *p* = 0.011), White race (HR 1.27, 95% CI: 1.16–1.40, *p* = 0.001), prostate cancer (HR 1.26, 95% CI: 1.10–1.45, *p* = 0.001), and breast cancer (HR 1.22, 95% CI: 1.04–1.44, *p* = 0.014). No significant interaction was observed for most subgroup variables.

### 3.7. Negative Control Outcomes

Analysis of negative control outcomes revealed no significant differences between treatment groups for cholelithiasis (HR 1.00, 95% CI: 0.84–1.19, *p* = 0.124), nephrolithiasis (HR 0.93, 95% CI: 0.79–1.09, *p* = 0.112), or burns (HR 0.62, 95% CI: 0.30–1.29, *p* = 0.889). These findings support the validity of the propensity score-matching approach and suggest minimal residual confounding in healthcare utilisation patterns (Appendix S6 in Supplementary Materials).

## 4. Discussion

In this large-scale target-trial-emulation study of patients aged ≥75 years with solid tumour–related bone metastases, we found that Denosumab was associated with a significantly lower risk of SREs and all-cause mortality than bisphosphonates. The greatest difference was observed for pathological fractures, whereas other SRE components did not differ significantly. These findings extend evidence from RCTs to a very old, real-world population that is underrepresented in pivotal trials and highly relevant to routine oncology and nephrology practice. Results were consistent across sensitivity and subgroup analyses. However, Denosumab was associated with a higher risk of hypocalcaemia, highlighting the need for routine biochemical monitoring and calcium and vitamin D supplementation in older adults.

Our findings regarding SRE prevention are consistent with the pivotal RCTs that demonstrated denosumab superiority over zoledronic acid [6,7,8]. The combined analysis of these trials reported a HR of 0.83 (95% CI: 0.76–0.90) in favour of Denosumab [15]. Despite these pivotal trials, their generalizability to older adults remains limited. The mean age of participants in these RCTs typically ranged from the late 50s to early 70s, and patients with advanced CKD were often excluded. Our observed HR of 1.15 (favouring Denosumab as the reference) corresponds to an equivalent HR of 0.87 when bisphosphonates are used as the reference. Our findings regarding SRE prevention are consistent with the pivotal RCTs that demonstrated denosumab superiority over zoledronic acid.

The observed mortality benefit associated with Denosumab is particularly noteworthy. While the pivotal trials did not demonstrate significant differences in overall survival, our findings suggest potential survival benefits in older patients. Several mechanisms could explain this observation. First, superior SRE prevention, especially pathological fracture with Denosumab, may translate into reduced morbidity and preserved functional status, thereby indirectly improving survival [3,16]. Second, the lack of renal toxicity of Denosumab may be particularly advantageous in older patients with age-related decline in renal function, owing to the high co-prevalence of CKD and skeletal disorders [17]. Third, differences in treatment adherence between monthly subcutaneous Denosumab and intravenous bisphosphonates requiring infusion centre visits may contribute to differential outcomes. From a mechanistic perspective, the superior efficacy of Denosumab in reducing SREs and mortality observed in this study may be further elucidated by its pharmacological properties. Since Denosumab circulates in the blood and neutralises RANKL in the extracellular fluid, it can reach and protect all bone surfaces, including high-turnover areas where tumour-induced RANKL expression is highest. This neutralisation effectively disrupts the vicious cycle between tumour cells and the bone microenvironment, whereby bone resorption releases growth factors that further stimulate tumour progression. Bisphosphonates, however, require active bone resorption for uptake and are restricted to the bone matrix. Furthermore, the rapid and potent suppression of the entire osteoclast population by Denosumab explains its association with a higher risk of hypocalcaemia, as it more abruptly halts the flux of calcium from the bone into the systemic circulation compared to the more localised action of bisphosphonates.

Consistent with this mechanism, hypocalcaemia was significantly more frequent among denosumab users, consistent with prior pharmacokinetic and clinical studies demonstrating an increased risk, particularly in patients with impaired renal function or vitamin D deficiency [10]. The significantly higher risk of hypocalcaemia aligns with its mechanism of action as a RANK ligand inhibitor, which suppresses bone resorption more potently than bisphosphonates [18,19]. In our cohort, hypocalcaemia occurred in 5.7% of denosumab users compared with 2.4% of bisphosphonate users, underscoring the importance of adequate calcium (e.g., 500–1000 mg daily) in combination with vitamin D_3_ (cholecalciferol) 400–800 IU daily, provided there are no contraindications. In patients at higher risk of hypocalcaemia—such as those with CKD, low baseline vitamin D levels, or malnutrition—active vitamin D analogues (e.g., calcitriol 0.25–0.5 μg daily) may be considered, with close biochemical monitoring. The influence of concomitant glucocorticoids, frequently used for symptom palliation and antiemetic prophylaxis, is another critical factor in skeletal health. Chronic glucocorticoid exposure accelerates bone loss by suppressing osteoblast activity and prolonging osteoclast survival, which may act synergistically with tumour-driven osteolysis to increase SRE risk. In our study, we addressed this by incorporating baseline corticosteroid use into the propensity score matching model to mitigate confounding by indication. However, due to database limitations, residual effects related to cumulative dosage and duration could not be fully captured. Despite these factors, the potent RANKL inhibition of Denosumab may offer superior protection against steroid-induced bone fragility compared to bisphosphonates, a benefit particularly relevant for older adults who are highly vulnerable to fracture-related morbidity.

Contrary to expectations given the known nephrotoxicity of bisphosphonates, we did not observe a significant difference in acute kidney injury between the treatment groups. This finding may reflect appropriate clinical practice in which patients with renal impairment are preferentially prescribed denosumab and bisphosphonate doses are adjusted based on renal function. Our exclusion of patients with severe renal impairment (eGFR <15) also reduced the population most vulnerable to bisphosphonate-related nephrotoxicity. The subgroup analyses also revealed important clinical implications. The treatment effect favouring Denosumab was most pronounced in patients aged ≥85 years, suggesting that very old individuals may derive particular benefit from Denosumab. Similarly, significant benefits were observed in patients with prostate and breast cancer, the two most common malignancies metastasising to bone. The lack of significant difference in Asian and Black populations warrants further investigation, though limited sample sizes in these subgroups may have reduced statistical power. Regarding gender distribution, our propensity score-matched cohorts achieved near-perfect balance between the sexes (SMD = 0.006). This ensures that the observed treatment benefits of Denosumab remained robust and were not confounded by the inherently higher skeletal vulnerability typically observed in female patients.

Our study has several strengths. First, the large sample size from a global federated network provides substantial statistical power and enhances generalisability. Second, the target trial emulation framework with PSM reduces confounding and various biases common in observational studies. Third, comprehensive sensitivity analyses and negative control outcomes support the robustness of our findings. Fourth, the focus on older patients addresses a significant evidence gap, as this population is underrepresented in clinical trials.

Several limitations should be acknowledged. First, as with any observational study, residual confounding cannot be entirely excluded despite rigorous PSM. However, E-values suggest moderate robustness to unmeasured confounding. Second, our study design is subject to potential immortal time bias, as patients were required to survive and remain SRE-free from the diagnosis of bone metastasis until 30 days after treatment initiation. This 30–365-day follow-up window was deliberately chosen to ensure causal temporality and to exclude events that were likely pre-existing or imminent before the treatments could achieve their pharmacological effects. While this survivor selection may limit generalizability to the most critically ill patients, the bias was applied symmetrically to both treatment groups through PSM, thereby preserving the validity of the relative comparison. Third, the TriNetX database may not capture all relevant clinical variables, including performance status, extent of bone metastases, and detailed cancer staging. Third, medication adherence could not be directly assessed, though the sensitivity analysis restricted to per-protocol patients showed consistent results. Fourth, the database relies on diagnostic and procedure codes, which may have variable accuracy across healthcare systems.

## 5. Conclusions

In conclusion, among patients aged ≥75 years with solid tumour-related bone metastases, Denosumab was associated with a lower risk of skeletal-related events—particularly pathological fractures—and reduced all-cause mortality compared with bisphosphonates. These findings extend evidence from randomised trials into a clinically vulnerable population and support Denosumab as a preferred bone-modifying agent in older adults, with appropriate monitoring for hypocalcaemia.

## Data Availability

The data presented in this study are available through the TriNetX platform. Restrictions apply to the availability of these data, which were used under licence for this study.

## Supplementary Materials

The following supporting information can be downloaded at https://www.mdpi.com/article/10.3390/life16020346/s1, [6,7,9,15,20,21].
