# Supplementary material for: Comparative Effectiveness and Safety of Denosumab Versus Bisphosphonates in Elderly Patients with Cancer Bone Metastases: A Target Trial Emulation Study
Source: Life (Basel). 2026 Feb 17;16(2):346. doi: 10.3390/life16020346 (PMC12941719; doi:10.3390/life16020346)
Supplement: Supplementary file 1 [file life-16-00346-s001.zip › life-4144190-supplementary.pdf]

SUPPLEMENTARY MATERIALS

Comparative Effectiveness and Safety of Denosumab versus Bisphosphonates in Elderly Patients (≥75 Years) with Cancer Bone Metastases: A Target Trial Emulation Study

Table of Contents

STROBE Checklist

TARGET Trial Emulation Checklist

Appendix S1. Target Trial Emulation Protocol

Appendix S2. Baseline Characteristics – Sensitivity Analysis: US Population (≥75 years)

Appendix S3. Baseline Characteristics – Sensitivity Analysis: Age ≥65 years

Appendix S4. Baseline Characteristics – Sensitivity Analysis: Per-Protocol

Appendix S5. Summary of All Outcomes Across Sensitivity Analyses

Appendix S6. Negative Control Outcomes

Appendix S7. Outcome Definitions and ICD-10/CPT Codes

Appendix S8. Medication Definitions and RxNorm Codes

Appendix S9. Comparison of Original and Revised Composite SRE Definitions

STROBE Statement Checklist

Item No.	Recommendation	Reported on Page/Section
**Title and Abstract**		
1	(a) Indicate the study's design with a commonly used term in the title or the abstract	Title, Abstract
1	(b) Provide in the abstract an informative and balanced summary of what was done and what was found	Abstract
**Introduction**		
2	Background/rationale: Explain the scientific background and rationale for the investigation being reported	Introduction, Para 1-4
3	Objectives: State specific objectives, including any prespecified hypotheses	Introduction, Para 5
**Methods**		
4	Study design: Present key elements of study design early in the paper	Methods: Study design
5	Setting: Describe the setting, locations, and relevant dates, including periods of recruitment, exposure, follow-up, and data collection	Methods: Study design; TriNetX Global Collaborative Network
6	(a) Cohort study: Give the eligibility criteria, and the sources and methods of selection of participants. Describe methods of follow-up	Methods: Study population
6	(b) Cohort study: For matched studies, give matching criteria and number of exposed and unexposed	Methods: Statistical analysis; Table 1
7	Variables: Clearly define all outcomes, exposures, predictors, potential confounders, and effect modifiers. Give diagnostic criteria, if applicable	Methods: Exposure definition, Outcomes, Covariates; Appendix 7-8
8	Data sources/measurement: For each variable of interest, give sources of data and details of methods of assessment (measurement). Describe comparability of assessment methods if there is more than one group	Methods: Data source (TriNetX); Appendix 7-8
9	Bias: Describe any efforts to address potential sources of bias	Methods: PSM methodology; Discussion: Limitations
10	Study size: Explain how the study size was arrived at	Results: Study population; Figure 1
11	Quantitative variables: Explain how quantitative variables were handled in the analyses. If applicable, describe which groupings were chosen and why	Methods: Statistical analysis; Covariates
12	(a) Statistical methods: Describe all statistical methods, including those used to control for confounding	Methods: Statistical analysis
12	(b) Describe any methods used to examine subgroups and interactions	Methods: Subgroup analyses; Figure 3

12	(c) Explain how missing data were addressed	Not explicitly addressed (limitation)
12	(d) Cohort study: If applicable, explain how loss to follow-up was addressed	Methods: Time-to-event analysis
12	(e) Describe any sensitivity analyses	Methods: Sensitivity analyses; Appendix 5
**Results**		
13	(a) Report numbers of individuals at each stage of study	Results: Study population; Figure 1
13	(b) Give reasons for non-participation at each stage	Figure 1 (exclusion criteria)
13	(c) Consider use of a flow diagram	Figure 1
14	(a) Descriptive data: Give characteristics of study participants and information on exposures and potential confounders	Results: Baseline characteristics; Table 1
14	(b) Indicate number of participants with missing data for each variable of interest	Not explicitly reported
14	(c) Cohort study: Summarise follow-up time	Methods: Outcome window 30-365 days; Figure 2 (numbers at risk)
15	Outcome data: Cohort study: Report numbers of outcome events or summary measures over time	Results: All outcomes; Table 2
16	(a) Main results: Give unadjusted estimates and, if applicable, confounder-adjusted estimates and their precision. Make clear which confounders were adjusted for and why they were included	Table 2 (after PSM); Methods: Covariates
16	(b) Report category boundaries when continuous variables were categorized	Methods: eGFR categories, age strata
16	(c) If relevant, consider translating estimates of relative risk into absolute risk for a meaningful time period	Table 2 (event rates reported)
17	Other analyses: Report other analyses done - e.g., analyses of subgroups and interactions, and sensitivity analyses	Results: Sensitivity analyses, Subgroup analyses; Appendix 2-5; Figure 3
**Discussion**		
18	Key results: Summarise key results with reference to study objectives	Discussion, Para 1; Key Points
19	Limitations: Discuss limitations of the study, taking into account sources of potential bias or imprecision. Discuss both direction and magnitude of any potential bias	Discussion: Limitations paragraph
20	Interpretation: Give a cautious overall interpretation of results considering objectives, limitations, multiplicity of analyses, results from similar studies, and other relevant evidence	Discussion: Para 2-6

21	Generalisability: Discuss the generalisability (external validity) of the study results	Discussion: Strengths paragraph (global network, large sample)
**Other Information**		
22	Funding: Give the source of funding and the role of the funders for the present study and, if applicable, for the original study on which the present article is based	Declaration: No external funding

Reference: von Elm E, Altman DG, Egger M, Pocock SJ, Gotzsche PC, Vandenbroucke JP. The Strengthening the Reporting of Observational Studies in Epidemiology (STROBE) statement: guidelines for reporting observational studies. Lancet. 2007;370(9596):1453-1457.

TARGET Trial Emulation (TTE) Checklist

Part 1: Target Trial Protocol Specification

Item	Target Trial Component	How Addressed in Study	Location in Manuscript
1	Eligibility Criteria	Age ≥75 years; solid tumour (ICD-10: C00-C80); bone metastasis (C79.5); first BMA after bone mets Dx; ≥12 months baseline records. Exclusions: multiple myeloma, prior comparator use, prior SRE, eGFR<15/ESRD, prior ONJ	Methods: Study population; Appendix 1
2	Treatment Strategies	Strategy A: Initiate bisphosphonates (zoledronic acid RxNorm:77655, pamidronate RxNorm:11473, clodronic acid RxNorm:3350). Strategy B: Initiate denosumab (RxNorm:993449, HCPCS:J0897)	Methods: Exposure definition; Appendix 8
3	Treatment Assignment	Patients assigned based on first BMA prescription after bone metastasis diagnosis (new-user design)	Methods: Exposure definition
4	Outcomes	Primary: Composite SRE (pathologic fracture M84.5x, spinal cord compression G95.2, radiation to bone CPT:77401-77425, surgery to bone CPT:22520-22525). Secondary: Individual SRE components, all-cause mortality. Safety: AKI, hypocalcaemia, ESRD	Methods: Outcomes; Appendix 7
5	Causal Contrast of Interest	Intention-to-treat: Effect of initiating bisphosphonates vs denosumab regardless of subsequent adherence. Per-protocol analysis also conducted.	Methods: Statistical analysis; Appendix 4
6	Follow-up Period	Time zero: Index date (first BMA prescription). End: 365 days after index, or outcome event, or death, or end of data availability. Outcome window: 30-365 days.	Methods: Study design; Appendix 1
7	Statistical Analysis	1:1 PSM using nearest-neighbour algorithm, caliper=0.1 SD. Cox proportional hazards regression for HRs with 95% CI. Kaplan-Meier survival curves with log-rank test.	Methods: Statistical analysis

Part 2: Emulation of Target Trial Components

Item	Target Trial Component	How Addressed in Study	Location in Manuscript
**A. Time Zero (Baseline) Definition**			
8	Time Zero Alignment	Index date = date of first BMA prescription after bone metastasis diagnosis. Same definition for both treatment groups.	Methods: Study design; Appendix 1
9	Immortal Time Bias Prevention	New-user design: Only patients initiating their first BMA included. Prevalent users excluded. Index date = first drug use.	Methods: Exclusion criteria; Discussion: Limitations
**B. Eligibility and Baseline Assessment**			
10	Baseline Window	12 months prior to index date used for covariate assessment and eligibility determination	Methods: Covariates; Appendix 1
11	Covariate Measurement	Demographics, cancer type, comorbidities, medications, laboratory values (BMI, eGFR) measured in 12-month baseline period	Methods: Covariates; Table 1
**C. Treatment Assignment and Confounding**			
12	Confounding Adjustment Method	Propensity score matching (1:1) with greedy nearest-neighbour algorithm, caliper = 0.1 SD of logit PS	Methods: Statistical analysis
13	Covariates for Adjustment	Age, sex, race, facility type, cancer type (breast, prostate, lung, kidney), CKD stages, eGFR, diabetes, heart failure, medications (corticosteroids, calcium, antineoplastics, endocrine therapy), BMI	Methods: Covariates; Table 1
14	Balance Assessment	Standardised mean differences (SMD) reported before and after matching. SMD <0.1 considered balanced. All variables achieved balance after PSM.	Results: Baseline characteristics; Table 1
**D. Follow-up and Outcome Assessment**			
15	Follow-up Duration	365 days maximum. Numbers at risk reported at 60-day intervals in KM curves.	Figure 2; Results
16	Outcome Ascertainment	ICD-10 and CPT codes from EHR data. Mortality from TriNetX Deceased Flag. Same methods for both groups.	Methods: Outcomes; Appendix 7
17	Censoring	ITT: At 365 days or administrative censoring. Per-protocol: Additional censoring at treatment discontinuation or switch.	Methods: Statistical analysis; Appendix 4
**E. Sensitivity and Robustness Analyses**			
18	Sensitivity Analyses	(1) US-only population; (2) Age ≥65 years; (3) Per-protocol analysis with continuous exposure requirement	Methods: Sensitivity analyses; Appendix 2-5
19	Subgroup Analyses	By age strata (75-79, 80-84, ≥85), race,	Results: Subgroup analyses; Figure

		facility type, eGFR level, cancer type, CKD, diabetes, heart failure	3
20	Negative Control Outcomes	Cholelithiasis, nephrolithiasis, burns analysed. All showed HR≈1.0 (non-significant), supporting absence of residual confounding.	Results: Negative control outcomes; Appendix 6
21	Unmeasured Confounding Assessment	E-values calculated for primary outcomes. SRE E-value=1.57; Mortality E-value=2.17, indicating moderate robustness.	Table 2; Results

Part 3: Potential Biases and Limitations Addressed

Potential Bias	Strategy to Address	Location
Immortal Time Bias	New-user design; index date = first drug use; excluded prevalent users	Methods; Discussion: Limitations
Selection Bias	Clear eligibility criteria; propensity score matching on baseline characteristics	Methods; Table 1; Figure 1
Confounding by Indication	PSM on baseline renal function; active comparator design; sensitivity analysis by eGFR strata	Methods; Discussion
Unmeasured Confounding	E-values calculated; negative control outcomes analysed (all HR≈1.0)	Table 2; Appendix 6
Differential Follow-up	Time-to-event analysis with Kaplan-Meier curves; numbers at risk reported	Figure 2; Methods
Outcome Misclassification	Validated ICD-10/CPT codes aligned with RCT definitions; outcomes defined a priori	Methods; Appendix 7

References for TARGET-TTE Framework:

1. Hernan MA, Robins JM. Using Big Data to Emulate a Target Trial When a Randomized Trial Is Not Available. Am J Epidemiol. 2016;183(8):758-764.

2. Hernan MA, Sauer BC, Hernandez-Diaz S, et al. Specifying a target trial prevents immortal time bias and other self-inflicted injuries in observational analyses. J Clin Epidemiol. 2016;79:70-75.

3. Matthews AA, Danaei G, Islam N, Kurth T. Target trial emulation: applying principles of randomised trials to observational studies. BMJ. 2022;378:e071108.

Appendix S1. Target Trial Emulation Protocol

Study Design and Data Source

This retrospective cohort study utilised the TriNetX Global Collaborative Network, a federated health research network encompassing electronic health records from over 193 million patients. We employed a target trial emulation framework with propensity score matching (PSM) to compare the effectiveness and safety of denosumab versus bisphosphonates (zoledronic acid, pamidronate, clodronic acid) in elderly patients (aged ≥75 years) with solid tumour bone metastases.

Inclusion Criteria

1. Age ≥75 years at index date
2. Solid tumour malignant neoplasm diagnosis (ICD-10: C00-C80, C7A, C7B)
3. Secondary malignant neoplasm of bone and bone marrow (ICD-10: C79.5, C79.51, C79.52)
4. First bone-modifying agent prescription after bone metastasis diagnosis
5. At least 12 months of healthcare records prior to index date

Exclusion Criteria

1. Multiple myeloma (ICD-10: C90) – different pathophysiology
2. Prior use of comparator drug – ensure treatment-naïve comparison
3. Skeletal-related event before index date (within 1 year) – focus on prevention
4. eGFR <15 or CKD Stage 5/ESRD (N18.5, N18.6, Z99.2) – contraindicated for bisphosphonates
5. History of drug-induced osteonecrosis of jaw (M87.18, M87.19)

Index Date and Follow-up

The index date was defined as the date of first bone-modifying agent prescription after bone metastasis diagnosis. Baseline period included 12 months prior to index date. Outcome window extended from 30 days to 365 days after index date.

Propensity Score Matching Methodology

Propensity scores were estimated using logistic regression with baseline covariates including demographics (age, sex, race, facility type), primary cancer type (breast, prostate, lung, renal), renal function (CKD stages 1-4, eGFR), comorbidities (diabetes mellitus, heart failure), cancer treatment (antineoplastic agents, endocrine therapy), baseline medications (corticosteroids, calcium supplements), and body mass index. A 1:1 nearest-neighbour matching was performed using a greedy algorithm with caliper = 0.1 SD. Covariate balance was assessed using standardised mean differences (SMD), with SMD <0.1 considered balanced.

Primary Outcome

The primary outcome was time to first Skeletal-Related Event (SRE), defined as a composite endpoint including: (1) Pathological fracture in neoplastic disease (M84.5x), (2) Spinal cord compression (G95.2), (3) Radiation therapy to bone (CPT: 77401-77425, 77385-77386), and (4) Surgery to bone (CPT: 22520-22525).

Statistical Analysis

Kaplan-Meier survival analysis was performed with log-rank test for between-group comparison. Cox proportional hazards regression was used to estimate hazard ratios (HR) with 95% confidence intervals (CI). Reference group was denosumab. E-values were calculated to assess robustness to unmeasured

confounding. Negative control outcomes (cholelithiasis, nephrolithiasis, burn) were analysed to detect residual confounding.

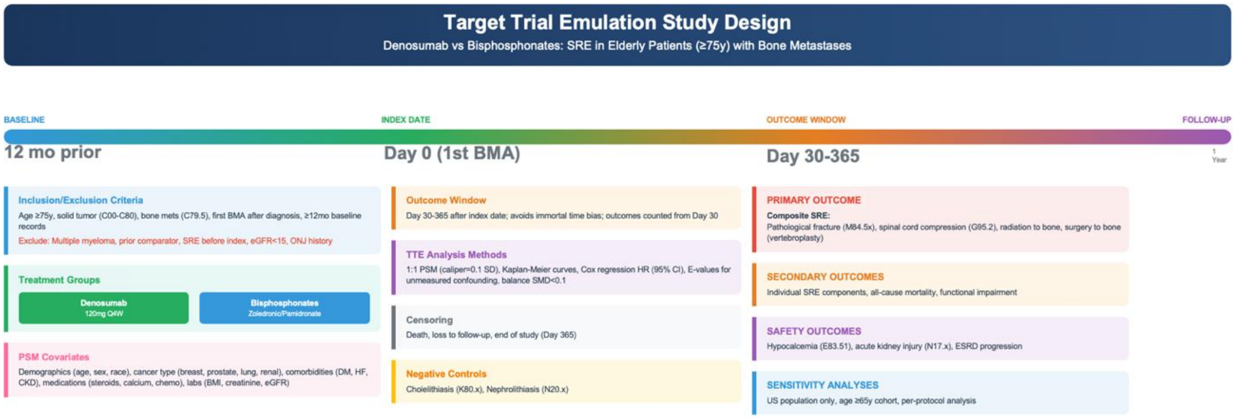

Appendix S2. Baseline Characteristics Before and After Propensity Score Matching – Sensitivity Analysis: US Population (≥75 years)

	Before Matching		After Matching			
	**Bisphosphonates**	**Denosumab**	**SMD**	**Bisphosphonates**	**Denosumab**	**SMD**
**N**	9,870	12,785		8,396	8,396	
Age, years (mean ± SD)	74.9 ± 6.9	76.6 ± 6.6	0.260	75.5 ± 6.9	75.5 ± 6.4	0.009
Male, n (%)	5,316 (55.2)	7,856 (61.4)	0.128	4,702 (56.0)	4,719 (56.2)	0.004
White, n (%)	7,546 (78.3)	9,875 (77.2)	0.026	6,591 (78.5)	6,701 (79.8)	0.032
Prostate cancer, n (%)	2,763 (28.7)	5,490 (42.9)	0.301	2,745 (32.7)	2,779 (33.1)	0.009
Breast cancer, n (%)	2,226 (23.1)	2,966 (23.2)	0.002	2,068 (24.6)	2,035 (24.2)	0.009
Lung cancer, n (%)	2,058 (21.4)	1,939 (15.2)	0.161	1,591 (18.9)	1,597 (19.0)	0.002
Diabetes mellitus, n (%)	1,929 (20.0)	2,235 (17.5)	0.065	1,569 (18.7)	1,553 (18.5)	0.005
eGFR, mL/min/1.73m² (mean ± SD)	74.9 ± 28.2	70.6 ± 26.2	0.159	74.1 ± 27.8	71.9 ± 26.6	0.083

Abbreviations: SMD, standardised mean difference; SD, standard deviation; eGFR, estimated glomerular filtration rate.

Appendix S3. Baseline Characteristics Before and After Propensity Score Matching – Sensitivity Analysis: Age ≥65 years

	Before Matching			After Matching		
	**Bisphosphonates**	**Denosumab**	**SMD**	**Bisphosphonates**	**Denosumab**	**SMD**
**N**	21,131	22,272		17,252	17,252	
Age, years (mean ± SD)	69.8 ± 8.6	72.2 ± 8.5	0.272	70.9 ± 8.5	70.9 ± 8.3	0.003
Male, n (%)	11,241 (54.4)	12,475 (56.0)	0.032	9,246 (53.6)	9,319 (54.0)	0.008
White, n (%)	13,881 (67.2)	15,499 (69.6)	0.051	11,836 (68.6)	11,913 (69.1)	0.010
Prostate cancer, n (%)	5,149 (24.9)	7,882 (35.4)	0.229	5,031 (29.2)	5,090 (29.5)	0.008
Breast cancer, n (%)	4,930 (23.9)	5,583 (25.1)	0.028	4,378 (25.4)	4,375 (25.4)	<0.001
Lung cancer, n (%)	4,561 (22.1)	4,137 (18.6)	0.087	3,615 (21.0)	3,528 (20.4)	0.012
Diabetes mellitus, n (%)	3,662 (17.7)	3,744 (16.8)	0.024	2,955 (17.1)	2,986 (17.3)	0.005
eGFR, mL/min/1.73m² (mean ± SD)	79.2 ± 30.3	74.8 ± 27.6	0.154	78.0 ± 29.8	76.0 ± 27.7	0.070

Abbreviations: SMD, standardised mean difference; SD, standard deviation; eGFR, estimated glomerular filtration rate.

Appendix S4. Baseline Characteristics Before and After Propensity Score Matching – Sensitivity Analysis: Per-Protocol

	Before Matching			After Matching		
	**Bisphosphonates**	**Denosumab**	**SMD**	**Bisphosphonates**	**Denosumab**	**SMD**
**N**	3,635	7,425		3,381	3,381	
Age, years (mean ± SD)	73.7 ± 6.8	76.2 ± 6.5	0.368	74.0 ± 6.6	74.3 ± 6.3	0.033
Male, n (%)	1,924 (55.1)	4,251 (57.3)	0.044	1,872 (55.4)	1,903 (56.3)	0.018
White, n (%)	2,467 (70.6)	5,384 (72.5)	0.043	2,422 (71.6)	2,448 (72.4)	0.017
Prostate cancer, n (%)	1,323 (37.9)	3,165 (42.6)	0.097	1,294 (38.3)	1,327 (39.2)	0.020
Breast cancer, n (%)	1,077 (30.8)	2,025 (27.3)	0.078	1,013 (30.0)	1,025 (30.3)	0.008
Chronic kidney disease, n (%)	155 (4.4)	845 (11.4)	0.260	155 (4.6)	148 (4.4)	0.010
eGFR, mL/min/1.73m² (mean ± SD)	76.7 ± 22.9	70.8 ± 24.2	0.251	76.5 ± 22.8	75.2 ± 23.1	0.054

Abbreviations: SMD, standardised mean difference; SD, standard deviation; eGFR, estimated glomerular filtration rate.

Appendix S5. Summary of All Outcomes Across Sensitivity Analyses

Hazard ratios (95% CI) for bisphosphonates vs denosumab (reference) across all sensitivity analyses.

**Outcome**	**Primary Analysis N=10,662/group**	**US Population N=8,396/group**	**Age ≥65 years N=17,252/group**	**Per-protocol N=3,381/group**
**Mortality**	1.41 (1.33-1.49) $P < 0.001$	1.41 (1.32-1.50) $P < 0.001$	1.43 (1.37-1.49) $P < 0.001$	1.17 (1.00-1.37) $P = 0.046$
**SRE**	1.15 (1.06-1.25) $P = 0.001$	1.18 (1.08-1.28) $P < 0.001$	1.15 (1.08-1.22) $P < 0.001$	1.23 (1.08-1.42) $P = 0.003$
Acute Kidney Injury	1.01 (0.92-1.11) $P = 0.816$	1.06 (0.97-1.17) $P = 0.208$	1.06 (0.99-1.14) $P = 0.110$	0.94 (0.78-1.13) $P = 0.485$
Hypocalcaemia	0.49 (0.43-0.57) $P < 0.001$	0.54 (0.47-0.63) $P < 0.001$	0.51 (0.46-0.57) $P < 0.001$	0.51 (0.40-0.66) $P < 0.001$

HR >1 indicates a higher risk in the bisphosphonate group. SRE, skeletal-related event.

Appendix S6. Negative Control Outcomes

Conditions without biological plausibility for differential association with bone-modifying agent choice. N=10,662 per group. Reference group: Denosumab.

**Outcome**	**Bisphosphonates Events, n (%)**	**Denosumab Events, n (%)**	**HR (95% CI)**	***P* value**
Cholelithiasis (K80.x)	238 (2.3)	283 (2.7)	1.00 (0.84-1.19)	0.98
Nephrolithiasis (N20.x)	220 (2.1)	300 (2.9)	0.93 (0.79-1.09)	0.34
Burn	11 (0.1)	21 (0.2)	0.62 (0.30-1.29)	0.20

All negative control outcomes show HR ≈ 1.0 (non-significant), supporting study validity and absence of systematic bias related to differential healthcare utilisation.

Appendix S7. Outcome Definitions and ICD-10/CPT Codes

Outcome	Definition	ICD-10/CPT Codes
**Skeletal-Related Events (SRE)**	Composite endpoint	See individual components
Pathological Fracture	Pathological fracture in neoplastic disease	M84.5x (primary), M84.4x (secondary)
Spinal Cord Compression	Other and unspecified cord compression	G95.2, G95.20, G95.29, G55
Radiation to Bone	Radiation treatment delivery	CPT: 77401-77407, 77412, 77417, 77424-77425, 77385-77386
Surgery to Bone	Vertebroplasty/Kyphoplasty	CPT: 22520-22525, 22310-22328
Functional Impairment	Immobilisation, Bed confinement	PCS2W3, Z74.01
Hypocalcaemia	Symptomatic hypocalcaemia	E83.51
Acute Kidney Injury	AKI requiring intervention	N17.x
ESRD	Progression to ESRD or dialysis	N18.6, Z99.2
All-cause Mortality	Death from any cause	TriNetX Deceased Flag

Appendix S8. Medication Definitions and RxNorm Codes

Cohort	Medication	RxNorm Code	Rationale
**Bisphosphonate**	Zoledronic acid	77655	FDA approved for bone mets
	Pamidronate	11473	FDA approved for bone mets
	Clodronic acid	3350	Used in Europe/Canada
**Denosumab**	Denosumab (Xgeva)	993449	120mg Q4W oncology dose
	Denosumab injection	HCPCS: J0897	Procedure code

Appendix S9. Comparison of Original and Revised Composite SRE Definitions

Outcome	Bisphosphonates Events, n (%)	Denosumab Events, n (%)	HR (95% CI)	P value	E-value
**Original composite SRE**	1,146 (10.7)	1,122 (10.5)	1.15 (1.06–1.25)	0.001	1.57
**Revised composite SRE***	1,054 (9.9)	1,037 (9.7)	*1.14 (1.05–1.24)*	*0.002*	*1.54*
Individual components:					
Pathological fracture	340 (3.2)	310 (2.9)	1.28 (1.10–1.49)	<0.001	1.88
Spinal cord compression	55 (0.5)	50 (0.5)	1.29 (0.88–1.89)	0.192	1.91
Radiation therapy to bone	635 (6.0)	650 (6.1)	1.10 (0.99–1.23)	0.082	1.42
Vertebral surgery	24 (0.2)	27 (0.3)	1.04 (0.60–1.81)	0.882	1.21
Functional impairment†	*92 (0.9)*	*85 (0.8)*	*1.27 (0.94–1.71)*	*0.118*	*1.85*

N = 10,662 per group. Reference group: Denosumab.

**Revised composite SRE excludes functional impairment, aligned with standard SRE definitions from pivotal RCTs (Stopeck 2010, Fizazi 2011, Henry 2011).*

†Removed from revised composite; reported separately as exploratory outcome.
Abbreviations: HR, hazard ratio; CI, confidence interval; SRE, skeletal-related event.

References

1. Stopeck AT, Lipton A, Body JJ et al. Denosumab compared with zoledronic acid for the treatment of bone metastases in patients with advanced breast cancer. J Clin Oncol 2010; 28: 5132–9.

2. Fizazi K, Carducci M, Smith M et al. Denosumab versus zoledronic acid for treatment of bone metastases in men with castration-resistant prostate cancer. Lancet 2011; 377: 813–22.

3. Henry DH, Costa L, Goldwasser F et al. Randomised, double-blind study of denosumab versus zoledronic acid in the treatment of bone metastases in patients with advanced cancer. J Clin Oncol 2011; 29: 1125–32.

4. Hong, S.; Youk, T; Lee ,S.J.; Kim, K.M Vajdic, C.M. Bone metastasis and skeletal-related events in patients with solid cancer: A Korean nationwide health insurance database study. PLoS One 2020; 15: e0234927.

5. Lipton A, Fizazi K, Stopeck AT et al. Superiority of denosumab to zoledronic acid for prevention of skeletal-related events: a combined analysis of 3 pivotal, randomised, phase 3 trials. Eur J Cancer 2012; 48: 3082–92.

6. TriNetX. TriNetX Global Collaborative Network. https://trinetx.com
